# Purification and characterization of a novel *β*-carotene-9′,10′-oxygenase from *Saccharomyces cerevisiae* ULI3

**DOI:** 10.1007/s10529-015-1872-7

**Published:** 2015-05-31

**Authors:** Tao Wei, Beilei Jia, Shen Huang, Kunpeng Yang, Chunxiao Jia, Duobin Mao

**Affiliations:** School of Food and Biological Engineering, Zhengzhou University of Light Industry, 5 Dongfeng Rd, Zhengzhou, 450002 People’s Republic of China

**Keywords:** *β*-Apo-10′-carotenal, *β*-Carotene, *β*-Carotene-9,10′-oxygenase, *Saccharomyces cerevisiae*, Strigolactone

## Abstract

**Objectives:**

A novel *β*-carotene-9,10′-oxygenase (ScBCO2) has been characterized from *Saccharomyces cerevisiae* ULI3 to convert *β*-carotene to *β*-apo-10′-carotenal, which is a precursor of the plant hormone strigolactone.

**Results:**

The ScBCO2 enzyme was purified to homogeneity by ammonium sulfate precipitation, Q sepharose and Superdex-200 chromatography. The molecular mass of the enzyme was ~50 kDa by SDS-PAGE. The purified ScBCO2 enzyme displayed optimal activity at 45 °C and pH 8. Tween 20 (1%, w/v), Trition X-100 (1%, w/v), Mg^2+^ (5 mM), Zn^2+^ (5 mM)_,_ Cu^2+^ (5 mM), Ca^2+^ (5 mM) or DTT (5 mM) increased in the activity by 3, 7, 14, 17, 23, 26 and 27%, respectively. ScBCO2 only exhibited cleavage activity towards carotenoid substrates containing two *β*-ionone rings and its catalytic efficiency (*kcat/Km*) followed the order *β*-carotene > *α*-carotene > lutein.

**Conclusion:**

ScBCO2 could be used as a potential candidate for the enzymatic biotransformation of *β*-carotene to *β*-apo-10′-carotenal in biotechnological applications.

**Electronic supplementary material:**

The online version of this article (doi:10.1007/s10529-015-1872-7) contains supplementary material, which is available to authorized users.

## Introduction

*β*-Carotene is an isoprenoid pigment occurring naturally in plants and fruits, and serves as the major dietary source of provitamin A. The in vivo cleavage of *β*-carotene can occur via two different biochemical pathways (Leuenberger et al. 2001; During and Harrison 2004). The first of these pathways, which is catalyzed by *β*-carotene 15,15′-monooxygenases (BCO1) in mammals (Lindqvist and Andersson 2002; Kim et al. 2011) and marine bacteria (Kim et al. 2009), involves the symmetrical cleavage of *β*-carotene into two molecules of retinal. The second pathway is an unusual and unsymmetrical cleavage process that occurs at the double bonds of *β*-carotene to produce long-chain (>C_20_) apocarotenoids, which are subsequently converted to retinal (Kim et al. 2009). Apocarotenoids function as antioxidants, hormones, volatile aromas and flavors, provitamin A and retinoid regulators (Walter and Strack 2011).

*β*-Apo-10′-carotenal (C27), which is one product of the asymmetric cleavage of *β*-carotene (apocarotenoids), is a precursor of strigolactone that acts as a novel plant hormone by promoting the seed germination and stimulating colonization of roots by symbiotic arbuscular mycorrhizal fungi (Akiyama et al. 2005). *β*-Apo-10′-carotenal could be converted from *β*-carotene by *β*-carotene-9,10′-oxygenase (BCO2) (Scherzinger and Al-Babili 2008), which cleave the C_9_-C_10_ double bond. Although BCO2 activities have been found in several higher organisms, including humans (Lindqvist and Andersson 2002), mice (Kiefer et al. 2001), ferrets (Hu et al. 2006) and cattle (Tian et al. 2009), there have been no reports in the literature to date pertaining to the isolation of an enzyme from a microorganism that is capable of converting *β*-carotene to *β*-apo-10′-carotenal. In this study, we have isolated and biochemically characterized a novel *β*-carotene-9,10′-oxygenase (ScBCO2) from *Saccharomyces cerevisiae* ULI3 and demonstrated that this enzyme could potentially be used for the enzymatic biotransformation of *β*-carotene to *β*-apo-10′-carotenal.

## Materials and methods

### Chemicals

*α*-Carotene, *β*-carotene, *γ*-carotene, zeaxanthin, lutein, lycopene and lutein were purchased from Pioneer Biotech Co., Ltd. (Xian, China). Unless otherwise stated, all of the chemicals used in the current study were purchased as the analytical grade.

### Organism and culture conditions

*Saccharomyces cerevisiae* ULI3 was isolated from fresh tobacco leaves, which were collected from Kunming, China. ScBCO2 was produced in complex medium (CM) containing (g/l): 1.5 g *β*-carotene, 3 g NaNO_3_, 0.5 g MgSO_4_, 1 g K_2_HPO_4_, 0.01 g FeSO_4_, 0.5 g KCl, 30 g sucrose and 3 g yeast extract power. The ULI3 strain was grown in a 500 ml flask containing 125 ml medium at 28 °C for 72 h with shaking at 150 rpm. The culture broth was collected and centrifuged at 6000×*g* for 20 min, and the supernatant was used as the crude enzyme for subsequent experiments.

### Purification of ScBCO2 from strain ULI3

The crude enzyme was fractionated by (NH_4_)_2_SO_4_ precipitation, where 60–80 % saturation with (NH_4_)_2_SO_4_ gave a precipitate, which was allowed to stand overnight. The precipitate was then collected by centrifugation at 12,000×*g* and suspended in 20 mM phosphate buffer (pH 8.0) before being loaded onto a Q-Sepharose column (1 × 20 cm^2^), which had been equilibrated with 20 mM phosphate buffer (pH 8.0). The proteins were eluted with a 100 ml of a linear gradient of NaCl from 0 to 1 M at 1 ml/min. Fractions were collected and tested for *β*-carotene degradation. Active fractions were pooled, filter concentrated, dialyzed in 50 mM phosphate buffer (pH 8) containing 200 mM NaCl and loaded onto a Superdex-200 (16/60) column, which had been equilibrated with 50 mM phosphate buffer (pH 8). All of the purification steps were carried out at 4 °C. The purified protein was analyzed by SDS-PAGE and the protein concentration was determined according to the Bradford method. Protein samples were stored in 20 mM phosphate buffer (pH 8.0) containing 25 % (v/v) glycerol at −80 °C.

### Enzyme assay

The reaction solution contained 200 mM NaCl, 12.5 μM Fe_2_SO_4_, 10 mM Tris (2-carboxylethyl) phosphine hydrochloride and 1.5 % (w/v) 1-*S*-octyl-*β*-D-thioglucopyranoside, as described previously (Kim et al. 2008). The enzyme and substrate solutions were mixed at 3:1 (v/v) and the reaction mixture was held at 37 °C for 60 min in 100 mM Tricine/KOH buffer (pH 8.0) containing 60 mM *β*-carotene, 1.5 % (w/v) Tween 40 and 0.3 U enzyme/ml. One unit of enzymatic activity was defined as the amount of enzyme required to liberate 1 mol *β*-apo-10′-carotenal per min under the standard conditions. The measurements were corrected for background hydrolysis in the absence of the enzyme.

### Analysis of HPLC and LC–MS

The ScBCO2-catalyzed conversion of *β*-carotene to *β*-apo-10′-carotenal was followed by HPLC using a Zorbax RX-C18 column (4.6 × 250 mm, 5 μm particle size, Agilent Technologies). The column was eluted with methanol/water (7:3 v/v) containing 0.1 % ammonium acetate (solvent B) and methanol (solvent A). The gradients were as follows: 100 % B to 0 % B over 12 min; 0 % solvent B for 8 min; and then 100 % A for 8 min at a flow rate of 0.5 ml/min. The reaction products were characterized by LC–MS. For quantification, a curve correlating peak area to moles of the reaction product was attained with *β*-apo-10′-carotenal as standard sample. A concentrated stock was prepared by dissolving *β*-apo-10′-carotenal in chloroform at 100 mg/l. This was diluted in chloroform to generate standards at 1, 2, 3, and 4 mg/l. The stocks were stored at −20 °C. Sample and standards were analyzed using an LTQ XL ultra-HPLC-ion trap MS equipped with an atmospheric pressure chemical ionization ion source (Thermo Electron Corp, USA). Mass fragmentation spectra were monitored for masses in the range of 50–800 atomic mass units on the LC–MS system. The chromatography conditions used for the LC–MS analysis were identical to those described above for the HPLC analysis.

### Characterization of ScBCO2

The effects of different temperatures, pH values, metal ions, organic solvents and reagents on the ScBCO2 activity were examined. The kinetic parameters were determined by assaying the purified enzyme at a variety of different substrate concentrations (10–800 μM) in three independent trials, and the corresponding *Km*, *Vmax* and *kcat* values were computed using Hanes–Woolf plots and the Michaelis–Menten equation.

### Analysis of purified enzyme

Protein bands were excised from SDS-PAGE, digested by trypsin and the sample was subjected to MALDI-TOF MS using an Applied Biosystems 4700 proteomics analyzer (Applied Biosystems, CA, USA). Data analysis was performed using the GPS Explorer software and MASCOT with the NCBI database.

## Results and discussion

### Purification and identification of ScBCO2

**Fig. 1:**
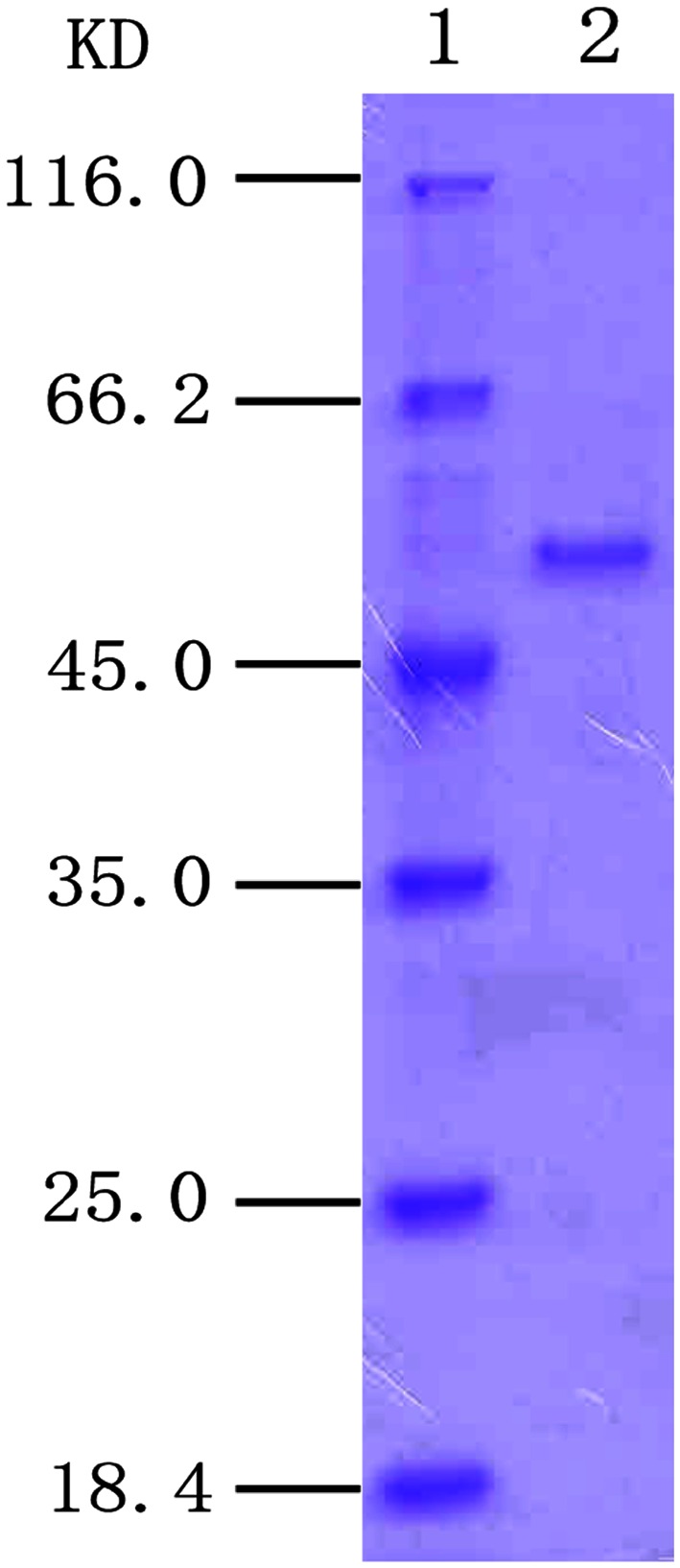
SDS-PAGE of the purified ScBCO2 enzyme from *S. cerevisiae* ULI3. *Lane 1* molecular weight standards; *lane 2* Superdex-200 chromatography product (purified enzyme)

The ScBCO2 enzyme with *β*-carotene cleavage activity was purified to homogeneity from the culture supernatant of the ULI3 strain. The purification procedure is shown in Table 1. The enzyme was purified 9.5-fold to give a yield of 10.8 % and a specific activity of 0.38 U/mg. The molecular mass of the purified enzyme was estimated to be 50 kDa by SDS-PAGE (Fig. 1), which is therefore less than those of the BCO2 enzymes isolated from mice (Kiefer et al. 2001), humans (Lindqvist and Andersson 2002), ferrets (Hu et al. 2006) and cattle (Tian et al. 2009). After being separated by SDS-PAGE, the ScBCO2 enzyme was analyzed by MALDI-TOF-MS, and the results showed that the peptide mass fingerprint of this enzyme did not fit with any of the other peptides in the database (data not shown). These results therefore indicated that the ScBCO2 enzyme from *S. cerevisiae* ULI3 could be a novel BCO2 oxygenase.

**Table 1:** Purification of ScBCO2 from the strain ULI3

Step	Total protein (mg)	Total activity (U)	Specific activity (U/mg)	Fold purification	Yield (%)
Crude cell extract	263	9.3	0.04	1	100
(NH_4_)_2_SO_4_ precipitation	92	5.2	0.06	1.5	55.9
Q Sepharose	21	1.9	0.09	2.25	20.4
Superdex-200	2.6	1.0	0.38	9.5	10.8

The values given are the average of three replication

### Identification of the reaction product of ScBCO2

**Fig. 2:**
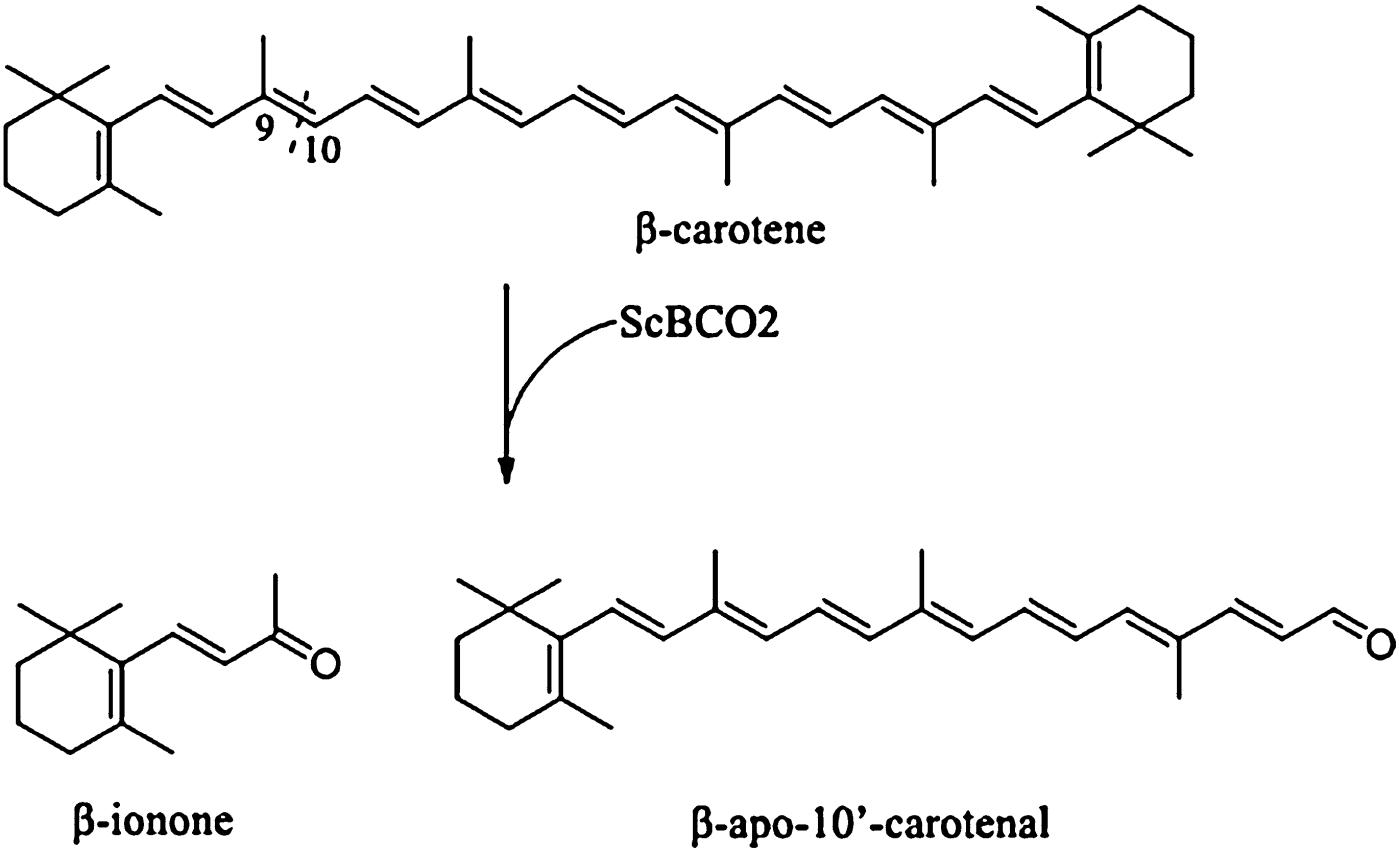
ScBCO2-catalyzed cleavage of *β*-carotene to give *β*-apo-10′-carotenal and *β*-ionone

LC–MS analysis of the enzyme reaction product revealed *m/z* values of 193 and 377, which corresponded to the quasimolecular ions of authentic *β*-ionone and *β*-apo-10′-carotenal samples, respectively (Supplementary Fig. 1a, b). These results therefore demonstrated that ScBCO2 could convert *β*-carotene to *β*-apo-10′carotenal and *β*-ionone as a consequence of its unusual cleavage activity towards the 9′,10′ double bond of *β*-carotene (Fig. 2). *β*-Apo-10′-carotenal is a precursor of the plant hormone strigolactone, which could promote seed germination and stimulate the colonization of plant roots by arbuscular mycorrhizal fungi (Akiyama et al. 2005). *β*-Ionone is a flavor and fragrance compound with fruity, violet-like characteristics and used in particular by foodstuff and beverage industries (Nacke et al. 2012). Some oxygenases in higher organisms exhibit high levels of unusual cleavage activity towards *β*-carotene, resulting in the production of *β*-apo-10′-carotenal (Kiefer et al. 2001; Lindqvist et al. 2005; Hu et al. 2006; Tian et al. 2009). There have, however, been no reports pertaining to the isolation and characterization of an enzyme from a microorganism that can convert *β*-carotene to *β*-apo-10′-carotenal. To the best of our knowledge, ScBCO2 therefore represents the first reported oxygenase to have been purified and characterized from a yeast source with unusual cleavage activity towards the production of *β*-apo-10′-carotenal.

### Effect of temperature and pH on enzyme activity

**Fig. 3:**
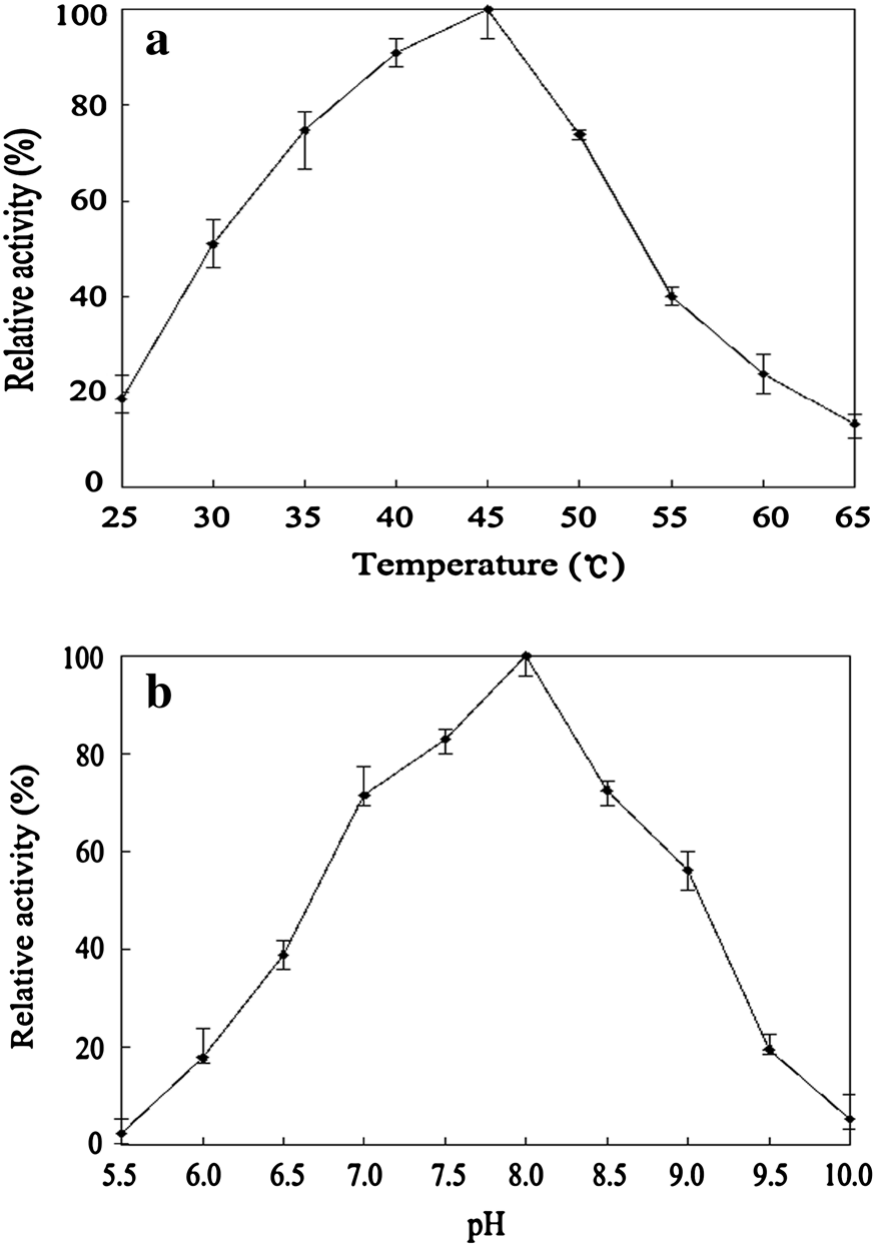
Temperature (**a**) and pH (**b**) optima of the ScBCO2 enzyme. **a** The optimum temperature for the ScBCO2 enzyme was determined using *β*-carotene as a substrate in 50 mM Tricine/KOH buffer (pH 8.0) at temperatures in the range of 25–65 °C. The activity at 45 °C (optimum temperature) was taken to be 100 % (0.3 U/mg). **b** The optimum pH for the ScBCO2 enzyme was determined at pH values in the range of 5.5–10.0 using *β*-carotene as a substrate for 60 min at 45 °C. The following buffer systems were used: 50 mM sodium acetate (pH 5.5 to 6.0), sodium phosphate (pH 6.5 to 8.0), Tris–HCl (pH 8.5 to 9.0) and N-cyclohexyl-3-aminopropanesulfonic acid (pH 9.5 to 10.0). The activity at pH 8.0 (optimum pH) was taken to be 100 % (0.3 U/mg). The relative activity was calculated by defining original activity as 100 %. The reported values are mean values of three independent experiments

The standard curve of the *β*-apo-10′-carotenal to quantify the product of the ScBCO2-catalyzed conversion of β-carotene is shown in Supplementary Fig. 2. The enzyme exhibited maximal activity ~45 °C with more than half of its maximal activity at 30–50 °C (Fig. 3a). It was optimally active at pH8 (Fig. 3b). Over 50 % of the maximal activity was maintained between pH 7 and 9. (Figure 3b).

### Effects of different metal ions, organic solvents and surfactants on the enzyme activity

Mg^2+^, Zn^2+^, Cu^2+^ or Ca^2+^ (5 mM) increased in the activity of ScBCO2 by 14, 17, 23 and 26 %, respectively, whereas Mn^2+^ or Fe^2+^ decreased the activity by 13 and 37 %, respectively (Table 2). The enzyme was not inhibited by 5 mM EDTA, which indicated that it was not a metalloprotein. The addition of Tween 20, Triton-X100 or DTT led to a slight increase in activity, whereas SDS (1 %) and urea (4 M) strongly inhibited the activity of ScBCO2 to less than 20 % of its optimal activity.

**Table 2:** Effect of various metals and chemicals on the cleavage activity of *β*-carotene of ScBCO2

Metals or inhibitors	Concentration	Relative activity (%)
None	–	100
Mg^2+^	5 mM	114 ± 1
Zn^2+^	5 mM	117 ± 3
Cu^2+^	5 mM	123 ± 2
Ca^2+^	5 mM	126 ± 4
Mn^2+^	5 mM	87 ± 5
Fe^2+^	5 mM	63 ± 2
Ni^2+^	5 mM	107 ± 3
K^+^	5 mM	101 ± 3
EDTA	5 mM	90 ± 2
DTT	5 mM	127 ± 4
Urea	4 M	14 ± 5
SDS	1 %(w/v)	1 ± 2
Tween 20	1 %(w/v)	103 ± 5
Triton X-100	1 %(w/v)	107 ± 3

Relative activity of the purified ScBCO2 was calculated by defining original activity as 100 % (0.3 U/mg). The values are means of three independent experiments

As shown in Table 3, the ScBCO2 enzyme was active in a number of organic solvents. Organic solvents are used in many industrial biocatalysts and therefore the effect of organic solvents on the activity of this enzyme is of special interest. ScBCO2 retained more than 70 % of its activity in 50 % (v/v) toluene, acetone, benzene, DMF or DMSO.

**Table 3:** Effect of organic solvents at 50 % (v/v) on the cleavage activity of *β*-carotene of ScBCO2

Organic solvents	Relative activity (%)^a^
None	100
Toluene	72 ± 3
Benzene	72 ± 1
Acetone	80 ± 4
2-Butanone	34 ± 3
Chloroform	43 ± 2
Dichloromethane	51 ± 5
DMF	76 ± 3
DMSO	83 ± 2

Organic solvents were added to reaction mixture containing purified enzymes ScBCO2, 50 mM Tricine/KOH buffer (pH 8.0), 60 mM *β*-carotene, 1.5 % (w/v) Tween 40, and 0.3 U enzyme/ml, then incubated at 45 °C for 60 min

^a^100 % activity = 0.3 U/mg. The values are means of three independent experiments

### Carotenoid substrate specificity of ScBCO2

Enzymatic activity was detected for *α*-carotene, *β*-carotene and lutein, but not for *γ*-carotene, zeaxanthin and lycopene (Supplementary Table 1). The *kcat*/*km* values for ScBCO2 followed the order *β*-carotene (77 min^−1^ mM^−1^) > *α*-carotene (49 min^−1^ mM^−1^) > lutein (10 min^−1^ mM^−1^) (Table 4). *α*-Carotene, *β*-carotene and lutein have two *β*-ionone rings in their structure, whereas *γ*-carotene and zeaxanthin contain only one *β*-ionone ring. In contrast, lycopene does not contain a *β*-ionone ring. Taken together, these results suggest that ScBCO2 has a stronger affinity and higher catalytic efficiency for substrates containing two *β*-ionone rings than any other substrates.

**Table 4:** Kinetic parameters of the oxygenase ScBCO2 for the cleavage of various carotenoids as substrates

Substrate	*k*_*cat*_ (min^−1^)	*K*_*m*_ (μM)	*k*_*cat*_ */K*_*m*_ (min^−1^ mM^−1^)
*β*-carotene	2.85 ± 0.3	37 ± 0.2	77 ± 3.1
*α*-carotene	2.35 ± 0.1	48 ± 0.3	49 ± 0.6
Lutein	1 ± 0.1	105 ± 0.3	10 ± 0.4

The values are means of three independent experiments

**Conclusion**: A novel *β*-carotene-9,10′-oxygenase from *S. cerevisiae* ULI3 has been purified and biochemically characterized for the first time. The molecular mass of the purified enzyme was ~50 kDa by SDS-PAGE; ptimum activities were at 45 °C and pH 8.0. The enzyme showed high cleavage activity towards *β*-carotene to form *β*-apo-10′-carotenal and *β*-ionone. These results therefore demonstrate that this enzyme could potentially be used for the synthesis of apocarotenoids in biotechnological applications.

## Electronic supplementary material

Supplementary material 1 (DOCX 167 kb)
