# Supplementary material for: Purification and characterization of a novel β-carotene-9′,10′-oxygenase from Saccharomyces cerevisiae ULI3
Source: Biotechnol Lett. 2015 May 31;37(10):1993–8. doi: 10.1007/s10529-015-1872-7 (PMC4565880; doi:10.1007/s10529-015-1872-7)
Supplement: Supplementary file 1 — Supplementary material 1 (DOCX 167 kb) [file 10529_2015_1872_MOESM1_ESM.docx]

**Supplementary Table 1**

Table 1 Carotenoid substrate specificity of ScBCO2

| Substrate | Specific activity (n mol min^-1^ mg^-1^) |
| --- | --- |
| *α*-carotene | 172±1.5 |
| *β*-carotene | 185±1.8 |
| lutein | 41±0.6 |
| *γ*-carotene | 3±0.3 |
| zeaxanthin | ND |
| lycopene | ND |

**Supplementary Fig. 1** LC-MS analysis of the reaction product catalyzed by ScBCO2. Mass spectrum of the typical fragment ions at m/z 193 (a) and 377 (b).

*
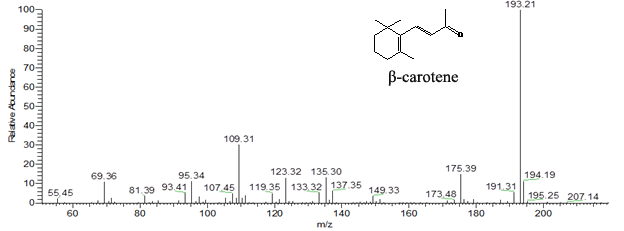
*

a

*
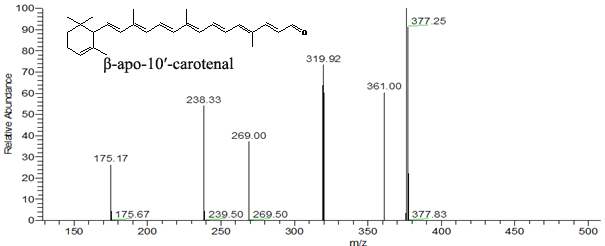
*

b

**Supplementary Fig. 2** The standard curve of the *β*-apo-10′-carotenal to quantify the product of the ScBCO2-catalyzed conversion of β-carotene.

*
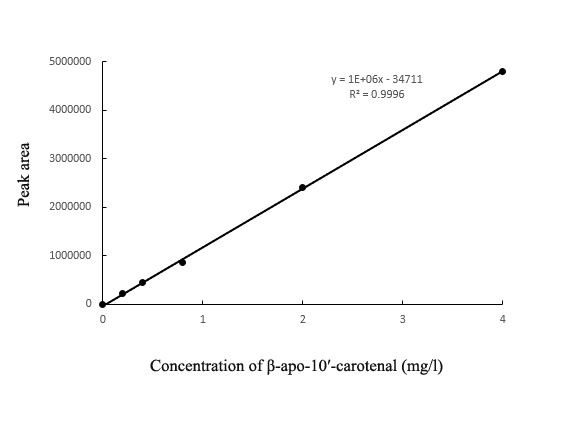
*
